# Letter from Dr. R. M. Swearingen, Texas State Health Officer, on “A Case of Septic-Salpingitis”

**Published:** 1885-08

**Authors:** R. M. Swearingen

**Affiliations:** Austin, Tex.


### CORRESPONDENCE.

### DR. McLAUGHLIN'S CASE OF SEPTIC SALPINGITIS.

*Letter from Dr. R. M. Swearingen, State Health Officer.*

AUSTIN, TEX., Aug. 1, 1885.

EDITOR DANIEL'S TEXAS MEDICAL JOURNAL:

*Dear Sir*:—I would respectfully direct your attention to "A Case of Septic Salpingitis Relieved by a New and Seemingly Simple Method" reported for the April number of the *Texas Courier Record of Medicine* by J. W. McLaughlin, M. D., of Austin, Texas.

In order to clearly understand the subject in all its bearings, I will quote the exact language used in the report, or so much thereof as may be necessary.

The Doctor says: "I delivered Mrs. Blank of a living child by means of the forceps. On the second day succeeding her confinement, she had a severe chill, followed by continued fever, sweating and pain, and tenderness of the uterus. * * I regarded the case as one of septic endometritis, caused by the presence in the womb of certain pathogenic bacteria. To destroy these micro-organisms, and control the disease, I used intra-uterine injections of mercuric bichloride, one grain to one pint of warm water, night and morning. The third day after this treatment was instituted, the fever left her; and I regarded my patient convalescent.

"All treatment was discontinued except vaginal washes, and the ordinary means of securing cleanliness. On the eleventh day following her confinement, she had another severe chill--fever rapidly ensued—temperature reaching to 105½. She had profuse sweatings, and complained of much pain and soreness in the region of the left ovary and oviduct. It seemed evident that some of the bacteria, from the first infection, had found a lodging place in her left oviduct—out of reach of germicidal washes—had there developed and multiplied until a septic salpingitis had resulted, and that death would speedily result from septicæmia, unless this local source of infection could be reached, and its cause destroyed. * * * I adopted the following method to reach and destroy the pathogenic bacteria which I believed to be present in her left fallopian tube, and endangering her life. So far as I know, this is a new departure in the treatment of such cases:

"I introduced a rubber catheter into the cavity of her womb; through this I slowly injected the solution of bi-chloride (1 gr. to 1 pint) until she complained that the uterus felt distended. I then withdrew the catheter, leaving about four ounces of the fluid in her uterine cavity. In the course of fifteen minutes she had violent pains in the pelvic region; these I controlled by hypodermic injection of sulphate of morphia. The next day she passed from her womb about four ounces of thick pus; the fever left her in a few days and she made a slow convalescence.

"The theory of the *modus operandi* of the injection is, that when the uterus was filled with fluid, rendered innocuous, and at the same time germicidal, it contracted violently, and forced part of the fluid into, and through, the fallopian tube. In this way the adhesions, or whatever obstructions existed at the mouth of the tube, were broken up, and it is supposed the fluid, a part of it, passed through the tube into the sac, and thus emptied it of its purulent contents, which was discharged per vaginam."

Very few of us have not used the intra-uterine washes; gynecologists (and there is an army of them now) resort to it frequently.

To meet the popular demand, or if you please, the exigences of the times, a double current catheter has been invented, to permit the escape of water. This recurrent channel was found to be very necessary, for an accumulation of water in the organ, very often brings on violent contractile pains. The Doctor was, of course, familiar with this phenomenon, and used the single rubber catheter, in order to insure the contractile pains.

According to the programme,the womb had to be temporarily converted into a kind of mitrailleuse, loaded with germicidal shot, and fired into a colony of bacteria,that could not be reached by any other means. The object to be attained was good, the plan of attack ingenious ; but could it be successful ?

Let us briefly analyze, and see. The pus was confined in the left fallopian tube. The mouth of that tube was closed, and had to be violently opened, before the bi-chloride solution could enter. When this was done, the injected solution came in contact with another fluid, that occupied and filled, to its utmost capacity, the narrow tube. Both fluids could not occupy the same space at the same time, nor could they possibly mix. When the solution was driven in at one end of the tube,the pus, by molecular force, must have been as violently driven out at the other end, or the tube was necessarily ruptured. In either contingency the two fluids would not commingle ; then how would it be possible for the one to destroy micro-organisms that existed in the other ?

There is one other fact to be considered. The mouth of the left fallopian tube was closed by either thickened walls, or adhesive inflammation, and full of pus.

In physics we are taught that "fluids seek channels which offer the least resistance." The right fallopian tube was in a normal condition, and had an opening or mouth that would not present such barriers to the escape of the solution,as were found to exist at the mouth of the other tube.

The conclusion, then, seems inevitable, that the bi-chloride solution, when pressed by the contracting uterus, would have passed out through the right fallopian tube, where no serious obstructions were met, instead of into the left tube, which was hermetically closed. Nature is very obstinate, and will not violate a fixed law to accommodate a theory.

If the germicidal solution, or any part of it, ever entered the abdominal cavity,it passed through the right fallopian tube,leaving the bacteria undisturbed.

As before stated, very few of us have not used the intra-uterine washes; probably a fewer number have seen the suffering that sometimes follows. It is safe to assume that those who have been witnesses, are satisfied with one rendition.

When the uterus is undergoing involution, the pain, referred to, is absolutely intolerable, and the subsequent prostration, profound.

Whether the physiological reduction gradually going on in the uterus, is materially interfered with or not, by changes so rapidly precipitated, I cannot say ; but I do know, that the cases make slow and imperfect recoveries, and that nervous prostration, and subinvolution very often follow in its wake.

When, in the judgment of a physician, it becomes imperative to invoke an agent so terrible and dangerous, in order to accomplish a special object, the object should be unmistakable, and the manner of its accomplishment, clearly defined. Can this be said of the new plan of treatment for septic salpingitis? Notwithstanding the glow of science, and glitter of technicals given to the report by its accomplished author, if we look at it through the subdued light of every day's experience, an affirmative answer cannot be given.

In closing, Mr. Editor, I beg leave to assure you, and the readers of your Journal,that only a professional interest inspires this letter. The paper criticised has been given the profession by one who justly ranks among the first in our State.

The theory advanced and treatment practiced is not a negative, halfway measure. It is either right or wrong, good or bad. If right and good, we should fall into line; if not, the fact should be demonstrated. Respect. Your Obed't Servant,

R. M. Swearingen.

